# Site-Specific Hypermethylation of SST 1stExon as a Biomarker for Predicting the Risk of Gastrointestinal Tract Cancers

**DOI:** 10.1155/2022/4570290

**Published:** 2022-02-12

**Authors:** Xiantong Dai, Xin Sun, Ying Wu, Zhi Lv, Zhanwu Yu, Yuan Yuan, Liping Sun

**Affiliations:** ^1^Tumor Etiology and Screening Department of Cancer Institute, And Key Laboratory of Cancer Etiology and Prevention in Liaoning Education Department, The First Hospital of China Medical University, Shenyang 110001, China; ^2^Key Laboratory of GI Cancer Etiology and Prevention in Liaoning Province, The First Hospital of China Medical University, Shenyang 110001, China; ^3^Department of Thoracic Surgery, Cancer Hospital of China Medical University, Liaoning Cancer Hospital & Institute, No. 44 Xiaoheyan Road, Shenyang, Liaoning 110042, China

## Abstract

**Background:**

DNA methylation is an important epigenetic modification in tumorigenesis, and similar epigenetic regulation mechanisms have been found in the gastrointestinal tract (GIT) cancers. *Somatostatin* (*SST*) has been confirmed to be expressed throughout the GIT. This study aimed to simultaneously explore the relationships between the *SST* methylation and the risks of three GIT cancers (esophageal cancer (EC), gastric cancer (GC), and colorectal cancer (CRC)) and to evaluate its diagnostic value.

**Methods:**

Differentially methylated regions (DMRs) of the *SST* gene, including TSS200, 1stExon, and the gene body, were identified in GIT cancers by The Cancer Genome Atlas (TCGA) database analysis. Further analyses were conducted in tissue samples of EC (*n* = 50), GC (*n* = 99), and CRC (*n* = 80). The *SST* methylation was detected by bisulfite-sequencing PCR (BSP), and the *SST* expression was detected by quantitative real-time reverse transcription-polymerase chain reaction (qRT-PCR).

**Results:**

In GIT cancers, DMR-related CpG islands were mainly located in the 1stExon. The methylation status of the *SST* 1stExon in the tumor tissues was significantly higher than that in the adjacent noncancerous tissues, and the methylation rates of the specific CpG sites were correlated with clinical phenotypes. The average methylation rate (AMR) of the *SST* 1stExon was negatively correlated with the *SST* gene expression in GC and CRC (both *P* < 0.001). For the diagnosis of GIT cancers, the combined detection of methylation at CpG sites +18 and +129 showed the highest area under the curve (AUC 0.698), with a sensitivity of 59.3% and a specificity of 72.8%.

**Conclusions:**

The site-specific hypermethylation of the *SST* 1stExon increases the risk of GIT cancers and might be a potential predictive marker for pan-GIT cancers.

## 1. Introduction

The gastrointestinal tract (GIT) is composed of tubular digestive organs that have highly similar organizational structures and many common features during tumorigenesis. The discoveries of common molecular events in GIT cancers may help us understand the pathways of tumorigenesis and identify effective biomarkers.

Epigenetic changes, such as DNA methylation and histone modification, are early events in the occurrence and development of GIT cancers [1]. The DNA methylation is the addition of a methyl group to the CG dinucleotide without an alteration in the DNA sequence, and it can often lead to gene silencing by inhibiting gene transcription [2]. Studies have shown that abnormal methylation could be associated with GIT cancers [3–6]. Abnormal DNA methylation is promising for clinical application as a noninvasive biomarker [7, 8].

The growth-hormone-release inhibitory hormone (somatostatin, *SST*) gene is located on chromosome 3q27.3 and contains 2 exons. It is a member of the cyclic peptide family and can be expressed throughout the body. Studies have shown that the *SST* can inhibit the release of numerous secondary hormones and affect both neurotransmission in the central nervous system and the proliferation of normal and tumorigenic cells. In the GIT, the *SST* is thought to regulate the inhibition of intestinal motility and gastric acid secretory activity [9]. Recent research found that *SST* could inhibit the occurrence and development of tumors directly or indirectly. The *SST* might inhibit growth factor-mediated mitosis signaling by blocking the autocrine/paracrine activity of growth-stimulating hormone and growth factors, thus inducing apoptosis [10]. It could also inhibit the secretion of somatotropin and exert antiangiogenic effects [11]. Our previous comprehensive bioinformatic analysis of aberrantly methylated differentially expressed genes showed that the *SST* is a hub gene in gastric cancer (GC) [12] and colorectal cancer (CRC) [13]. However, the patterns of the *SST* methylation and expression in esophageal cancer (EC) are not clear.

Therefore, in the present study, we detected and analyzed the associations between the methylation status of the specific CpG sites in the *SST* 1stExon and the cancer risk, clinicopathological features, and gene expression profiles of esophageal cancer, gastric cancer, and colorectal cancer. The results provided valuable insights for the study of biomarkers for pandigestive tract carcinomas.

## 2. Materials and Methods

### 2.1. Patients and Specimens

Matched samples of the tumor tissues and tumor-adjacent noncancerous tissues were collected from the patients with GIT cancers who underwent surgical resection without preoperative physical or chemical therapies at the First Hospital of China Medical University and the Cancer Hospital of China Medical University between January 2013 and May 2018; 50 patients with EC, 99 patients with GC, and 80 patients with CRC were included. Detailed clinical data, including sex, age, pathological classification, pTNM classification, lymph node invasion status, vascular tumor emboli status, perineural invasion status, and depth of infiltration, were collected from the medical records of the hospital. Tissue samples were placed in RNAlater solution (RNAlater™ Stabilization Solution, Thermo Fisher Scientific, Waltham, MA, USA) immediately after surgery and subsequently frozen at –80°C until RNA extraction.

The current study was approved by the Human Ethics Review Committee of the First Hospital of China Medical University (Shenyang, China) and the Cancer Hospital of China Medical University (Shenyang, China). Each participant in the study signed an informed consent form.

### 2.2. Data Processing

Public DNA methylation data and annotations were acquired from The Cancer Genome Atlas (TCGA) database. The methylation data (beta-value matrix) of three GIT cancer cohorts comprising 16 paired EC, 2 paired GC, and 38 paired CRC samples were downloaded from UCSC Xena (https://xenabrowser.net/datapages/). Differentially methylated regions (DMRs) in the tumor tissues and normal tissues were identified using the bumphunter algorithm with the R (version 4.0.3) package ChAMP [14]. Only regions containing more than seven probes were defined as DMRs, with the significance threshold set at <0.05. MethPrimer 2.0 was used to analyze the locations of CpG islands in the *SST.*

### 2.3. DNA Extraction

The SDS-phenol extraction method was used to extract the tissue DNA. The tissue (0.1–0.2 g) was shredded and ground. Then, 400 *μ*l of TE, 25 *μ*l of 10% SDS, and 10 *μ*l of 20 mg/ml PK enzyme were added. The samples were mixed well and incubated at 55°C in a water bath for 2 h. Proteins were removed using a phenol, chloroform, and isoamyl alcohol mixture. DNA was precipitated with NaAc and absolute ethanol and resuspended in the TE solution [12].

### 2.4. Bisulfite Sequence Polymerase Chain Reaction (BSP)

The *SST* methylation was detected using BSP (bisulfite-sequencing polymerase chain reaction). Genomic DNA sulfite modification was performed using a Zymo DNA Methylation-Gold Kit (Zymo). The MethPrimer 2.0 was used to design the BSP primers. The outer primer sequences were forward, 5′- GTGTAATTGAGTGTGTATGTGTGGGAG -3′ and reverse, 5′- ACAACAACCAAAAACTTCTACAAAAACTAAC -3′. The inner primer sequences were forward, 5′- AATGTGTATGTTTATAGTATTGAGTGA -3′ and reverse, 5′- AACACAACCCAAAACCAA -3′. The thermal cycling program for PCR was as follows: denaturation at 94°C for 10 min; 40 cycles at 94°C for 20 s, 55°C for 30 s, and 72°C for 30 s; and a final extension step at 72°C for 10 min. The reactions were stored at 4°C. Agarose gel electrophoresis was used to check the quality of the PCR-amplified products. Sanger sequencing was used to determine the methylation status. The values of the C and T signals were read for each CpG site. The methylation rate of each site was calculated according to the equation Meth% = C/(C + T)∗100%.

### 2.5. Total RNA Extraction and Reverse Transcription

Total RNA was extracted from collected tissues using TRIzol (TaKaRa, Dalian, China) according to the protocol. Rice-sized fragments of mucosa were digested with TRIzol. Proteins were removed with chloroform. DNA contamination was removed using DNase I (Sangon Biotech, Shanghai, China). The operations were as follows: First, 16 *μ*l of RNA, 2 *μ*l of reaction buffer (10x) with MgCl_2_, and 2 *μ*l of DNase I, RNase-free (1 U/*μ*l) were combined, and the mixture was then incubated at 37°C for 30 min. Next, DNase I was inactivated by adding 2 *μ*l of 5 mM EDTA into the reaction system. Finally, the mixture was incubated at 65°C for 10 min. RNA was precipitated with isopropanol and washed with 75% ethanol. After the ethanol was evaporated, the RNA was dissolved in DEPC water. The concentration and purity of the RNAs were measured with a NanoDrop spectrophotometer (Thermo Scientific, America). Reverse transcription was carried out using the PrimeScript RT Master Mix (TaKaRa, Dalian, China) and oligo (dT) primers (TaKaRa, Dalian, China) according to the manufacturer's instructions. Each reaction mixture contained 1000 ng of total RNA and 4 *μ*l of the 5X PrimeScript RT Master Mix, and RNase-free water was then added to a total volume of 20 *μ*l. The mixtures were incubated at 37°C for 15 min (reverse transcription) and 85°C for 5 sec (for heat inactivation of reverse transcriptase) and were then held at 4°C.

### 2.6. Real-Time Quantitative PCR

The expression levels of the *SST* and an internal control gene (GAPDH) were determined by the real-time quantitative PCR (qRT–PCR) using TB Green Premix Ex Taq (TaKaRa). The primer sequences were as follows: *SST* forward, 5′- CTGAACCCAACCAGACGGAG -3′; *SST* reverse, 5′- GCCATAGCCGGGTTTGAGTT -3′; GAPDH forward, 5′- CCATCTTCCAGGAGCGAGATCCCT -3′; and GAPDH reverse, 5′- CCTGCAAATGAGCCCCAGCC -3′. The thermal cycling conditions were as follows: 95°C for 30 s; 40 cycles at 94°C for 30 s, 56°C for 20 s, and 72°C for 10 s; and holding at 4°C. Melting curve analysis was used to verify specificity and exclude nonspecific products and primer dimers. No-template controls were included in each experiment, and duplicate reactions were performed. Relative quantification of the SST expression was performed using the 2-*Δ*Ct method, and the expression level of SST was normalized to that of GAPDH in each sample using the equation ΔCt = Ct target − Ct GAPDH. The 2-*Δ*Ct values based on the *Δ*Ct values were considered the relative expression levels.

### 2.7. Statistical Analysis

Statistical analysis was performed using the IBM SPSS Statistics 23 software and R (version 4.0.2). Paired Student's *t* test was used to compare the differences in methylation and mRNA expression between cancer and control tissues. ANOVA was used to compare the relationships of methylation rates with tumor biological behaviors. Spearman rank correlation analysis was used to analyze correlations between methylation and mRNA expression levels. Receiver operating characteristic (ROC) curve analysis was used to evaluate the diagnostic efficacy and the area under the curve (AUC) values. The sensitivity (SEN), specificity (SPE), and Youden index (YD) were also calculated. Multivariate logistic regression was used to build appropriate diagnostic models. *P* < 0.05 was considered statistically significant.

## 3. Results

### 3.1. DMRs of the *SST* Gene in Three GIT Cancers

**Figure 1:**
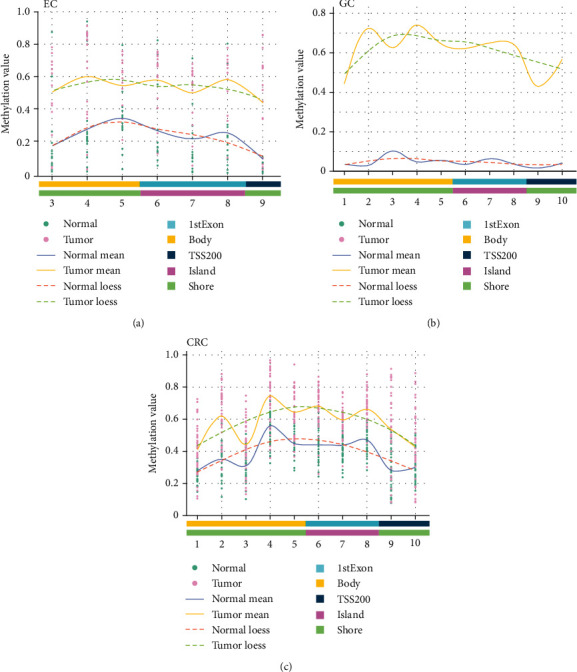
The DMRs in the *SST* gene in GIT cancers from the TCGA database. (a) The methylation status of DMRs in EC. (b) The methylation status of DMRs in GC. (c) The methylation status of DMRs in CRC. The *x*-axis shows the number of the CpG site.

DMR-related CpG sites were located in the TSS200, 1stExon, and gene body regions, while CpG islands were mainly located in the 1stExon; the related characteristics of CpG sites in the *SST* DMR are shown in Supplementary Table S1. The methylation statuses of the DMRs in the three GIT cancers were similar; that is, the methylation level at each CpG site in cancer tissues was higher than that in the adjacent noncancerous tissues (Figure 1).

### 3.2. *SST* 1stExon Methylation in GIT Cancers

**Figure 2:**
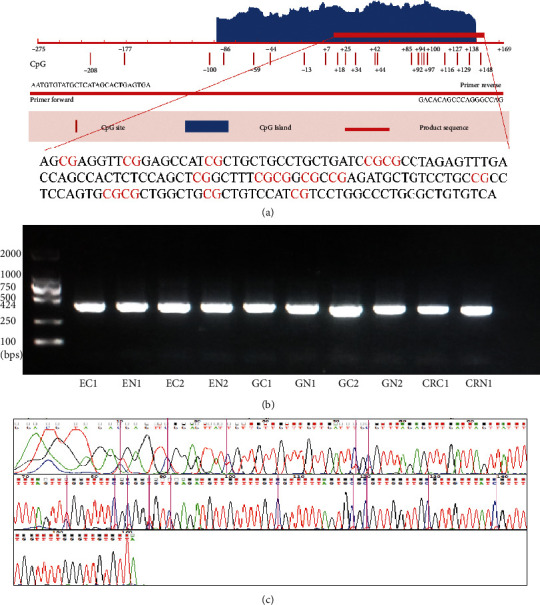
Amplified sequence of the *SST* 1stExon and Sanger sequencing results. (a) The amplified sequence was mainly located in the CpG island in *SST* 1stExon. (b) Agarose gel electrophoresis bands of PCR-amplified products after bisulfite modification. The left lane shows the DNA marker. EC, GC, and CRC represent tumor tissues, and EN, GN, and CRN represent tumor-adjacent noncancerous tissues. (c) The Sanger sequencing results for the PCR products. The purple bars represent the CpG sites. The blue line shows the signal intensity of the methylated C bases, and the red line at the corresponding positions shows the signal intensity of the unmethylated T bases.

The TSS of the *SST* was defined as 1 bp, and the 1stExon sequence extended from 1 bp-241 bp and contained 21 CpG sites. Fifteen CpG sites located in the CpG island in the 1stExon were identified by PCR product sequencing (Figure 2(a)). The results of agarose gel electrophoresis of the PCR amplification products are shown in Figure 2(b), and the Sanger sequencing results are shown in Figure 2(c).

**Figure 3:**
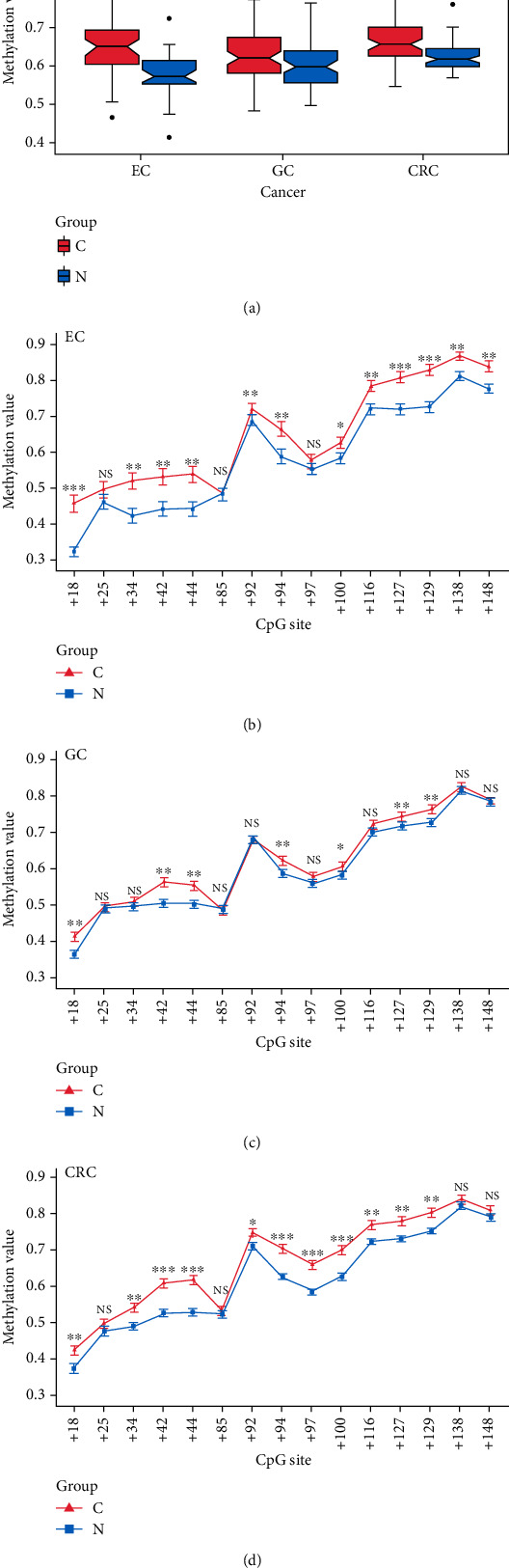
AMR and methylation status of each CpG site in the *SST* 1stExon. (a) AMR in the three cancers. (b) Methylation status of each CpG site in EC. (c) Methylation status of each CpG site in GC. (d) Methylation status of each CpG site in CRC. EC: esophageal cancer; GC: gastric cancer; CRC: colorectal cancer; NS: nonsignificant; C: tumor tissues; N: tumor-adjacent noncancerous tissues; ^∗^*P* < 0.05; ^∗∗^*P* < 0.01; ^∗∗∗^*P* < 0.001; ^∗∗∗∗^*P* < 0.0001.

The average methylation rate (AMR) and the methylation status of each site in the *SST* 1stExon in the three cancers are shown in Figures 3(a)–3(d) and Supplementary Tables S2–S4.

In the 42 cases of EC, the AMR and the methylation rates of the twelve CpG sites in the cancer tissues were significantly higher than those in the control tissues (all *P* < 0.05).

In the 99 cases of GC, the AMR and the methylation rates of the seven CpG sites in the cancer tissues were significantly higher than those in the control tissues (all *P* < 0.05).

In the 70 cases of CRC, the AMR and the methylation rates of the thirteen CpG sites in the cancer tissues were significantly higher than those in the control tissues.

The combined analysis of CpG site methylation showed that seven CpG sites (+18, +42, +44, +94, +100, +127, and +129) were cohypermethylated sites in all three cancers (Table 1).

### 3.3. Correlations between the *SST* 1stExon Methylation and Clinical Phenotypes

We further analyzed the relationships between the *SST* 1stExon methylation and clinical phenotypes, and the results are shown in Table 2.

In EC, compared with those in the negative vascular tumor emboli group, the methylation rates of site +127 (0.861 ± 0.071 vs. 0.794 ± 0.090, *P* = 0.021) and site +129 (0.878 ± 0.068 vs. 0.813 ± 0.093, *P* = 0.038) were significantly higher than those in the positive group. When considering the depth of infiltration, the methylation rate of site +85 in the muscular layer group (0.422 ± 0.105, *P* = 0.041) was significantly lower than those in the serous layer group (0.507 ± 0.117) and the mucosa and submucosa group (0.534 ± 0.172). For site +18, the methylation rate in the poor differentiation group (0.616 ± 0.137, *P* =0.014) was higher than those in the moderate differentiation group (0.432 ± 0.153) and the high differentiation group (0.442 ± 0.145).

In GC, the methylation rate of site +25 in the positive lymph node metastasis group (0.477 ± 0.092) was significantly lower than that in the negative lymph node metastasis group (0.536 ± 0.103, *P* = 0.013).

In CRC, the methylation rate of site +94 in the serous layer group (0.718 ± 0.098) was significantly higher than that in the muscular layer group (0.649 ± 0.129, *P* = 0.025).

### 3.4. *SST* Expression Levels in GIT Cancers

**Figure 4:**
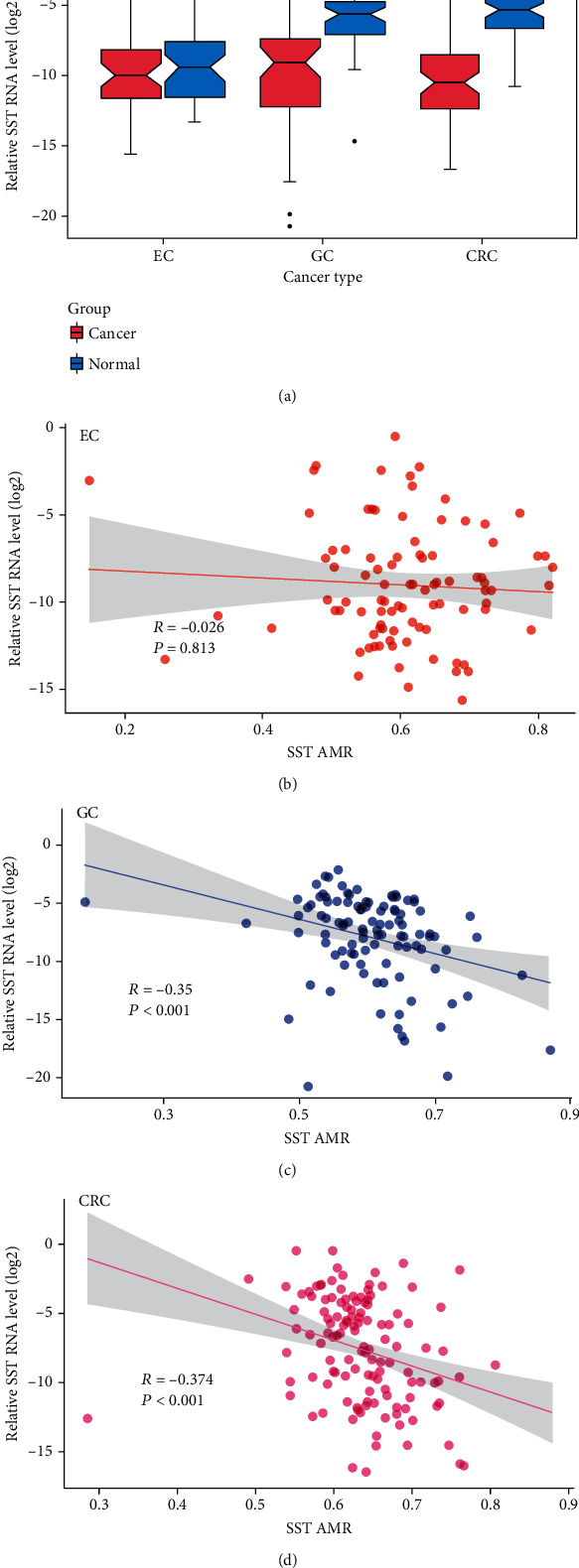
Differential *SST* expression and its correlations with methylation in GIT cancers. (a) Differential *SST* expression in GIT cancers. C: tumor tissues; N: tumor-adjacent noncancerous tissues. (b) Correlation between the *SST* expression and the *SST* AMR in EC. (c) Correlation between the *SST* expression and the *SST* AMR in GC. (d) Correlation between the *SST* expression and the *SST* AMR in CRC. NS: nonsignificant; ^∗^*P* < 0.05; ^∗∗^*P* < 0.01; ^∗∗∗^*P* < 0.001; ^∗∗∗∗^*P* < 0.0001.

In the 50 cases of EC, there was no significant difference in the *SST* expression between the cancer and adjacent noncancerous tissues (0.0167 ± 0.0455 vs. 0.033 ± 0.1061, *P* = 0.32). In the 52 cases of GC, the *SST* expression was significantly lower in the cancer tissues than in the adjacent noncancerous tissues (0.0086 ± 0.0176 vs. 0.0318 ± 0.0404, *P* < 0.001). In the 65 cases of CRC, the *SST* expression was significantly lower in the cancer tissues than in the adjacent noncancerous tissues (0.0098 ± 0.0263 vs. 0.0819 ± 0.1372, *P* < 0.001) (Figure 4(a)).

Then, we calculated the correlations between the *SST* 1stExon methylation and *SST* expression in GIT cancers.

The AMR of the *SST* 1stExon was not significantly correlated with the *SST* expression in the EC group, but it was negatively correlated with the *SST* expression in the GC and CRC groups (Figures 4(b)–4(d)). In the GC group, except at CpG sites +25 and +85, the *SST* methylation and expression were negatively correlated (*P* < 0.05). In the CRC group, except at CpG sites +25, +85, and+148, the *SST* methylation and expression were negatively correlated (*P* < 0.05) (Supplementary Table S5).

### 3.5. The Diagnostic Efficacy of the *SST* 1stExon Methylation for GIT Cancers

**Figure 5:**
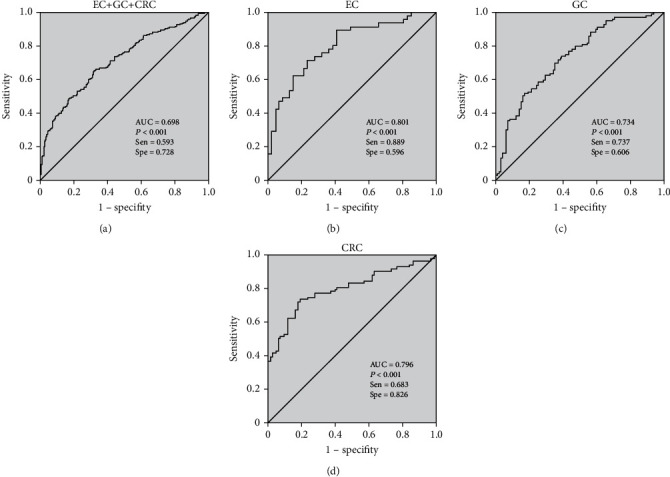
ROC of diagnostic models for GIT cancers. (a) ROC of CpG sites +18 and +129 for EC + GC + CRC. (b) ROC of CpG site +129 for EC. (c) ROC of CpG sites +18, +42, +44, +127, and +129 for GC. (d) ROC of CpG sites +44 and +94 for CRC. SEN: sensitivity; SPE: specificity.

ROC curves for the diagnosis of each individual cancer and the diagnosis of pan-GIT cancers were drawn based on codifferential CpG sites. For GIT cancers, a combination of two CpG sites (+18 and +129) had the largest AUC (0.698), with a SEN of 59.3% and a SPE of 72.8%. Among the individual cancers, CpG site +129 had the best diagnostic efficacy, with an AUC of 0.801, a SEN of 88.9%, and a SPE of 59.6% in EC. In GC, the CpG sites +18, +42, +44, +127, and +129 had the best diagnostic efficacy, with an AUC of 0.734, a SEN of 73.7%, and a SPE of 60.6%. In CRC, CpG sites +44 and +94 had the best diagnostic efficacy, with an AUC of 0.796, a SEN of 68.3%, and a SPE of 82.6% (Figure 5).

## 4. Discussion

It is commonly believed that the abnormal DNA methylation, one of the most important epigenetic alterations, is often related to CpG island activity and could regulate gene expression in tumorigenesis [15]. Previous studies have shown the abnormal DNA methylation of the *SST* in the head and neck squamous cell carcinoma [16], pancreatic ductal adenocarcinoma [17], and esophageal cancer [18] and found downregulation of the *SST* in the GC tissues [19, 20]. However, the role and inactivation mechanisms of *SST* methylation have not been thoroughly investigated in GIT tumorigenesis. Here, for the first time, we systematically studied the relationship between the methylation of the *SST* 1stExon CpG sites and the risk of GIT cancers in TCGA and tissue samples and further evaluated the diagnostic efficacy of the *SST* methylation.

First, our bioinformatic analysis results based on the TCGA database showed that the *SST* methylation level—especially in 1stExon, which is rich in CpG islands—in the tissues of the three GIT cancers was significantly higher than that in the corresponding adjacent noncancerous tissues. These results suggested that the abnormal *SST* methylation might be a potential biomarker to identify these three GIT cancers at the same time.

Then, we used tissue samples to validate whether the *SST* methylation plays a role in these three GIT cancers. In our study, we mainly focused on the *SST* CpG sites in the CpG island in the 1stExon region. We used the BSP method to detect the methylation of CpG sites in the *SST* gene in GIT cancers. The results showed that both the *SST* methylation level at a single CpG site and the AMRs in the three GIT cancer tissues were significantly higher than those in the corresponding adjacent noncancerous tissues, and the trend was the same in the TCGA database. Further analysis focused on the *SST* methylation at each CpG site, which was different from that identified in the previous studies. The previous studies mainly elucidated the role of the *SST* methylation based on the AMR [16]. We found that the cohypermethylated sites in all three cancers were CpG sites +18, +42, +44, +94, +100, +127, and +129, which might play important roles in GIT cancers. In addition, we analyzed the relationships between the *SST* methylation and clinical phenotypes, and the results showed that methylation of the *SST* CpG sites in 1stExon could be related to differentiation status, lymph node metastasis status, vascular tumor thrombus status, and infiltration depth, suggesting that hypermethylation of the CpG sites in the *SST* 1stExon region may influence the tumor biological behavior of GIT cancers. In the future, an in-depth functional study of *SST*-specific CpG sites is expected to reveal the molecular mechanism of abnormal *SST* methylation involved in the development of gastrointestinal tumors. Regarding *SST* expression, we found that the *SST* expression was markedly downregulated in GC tissues, and this result was consistent with the studies conducted by Zhang et al. [20] and Wang et al. [19]. We also observed that the *SST* expression was significantly decreased in CRC tissues, a finding that was also supported by Leiszter et al. [21]. However, there was no significant difference in the *SST* expression between the EC tissue and adjacent noncancerous tissue. We speculated that this might be due to the low *SST* transcript level in esophageal tissue. Therefore, the relationship between *SST* and EC risk needs further study.

Recent studies have demonstrated that aberrant DNA methylation could be an epigenetic regulator of gene expression [22, 23]. In our study, the results showed that the AMR of *SST* was significantly negatively correlated with the *SST* expression level in GC and CRC. Misawa et al. also observed hypermethylation and downregulated expression of *SST* in the head and neck squamous cell carcinoma [16], and these results showed that the abnormal *SST* methylation might participate in the occurrence and development of GC and CRC by regulating *SST* expression. Moreover, we found that methylation at twelve CpG sites was significantly negatively correlated with the *SST* expression in GC, while in CRC, thirteen CpG sites exhibited such a correlation. Recent studies have shown that methylation usually occurs at the CpG sites in CpG islands. The addition of a new methyl group to the 5-carbon atom of cytosine can cause a corresponding change in the chromatin conformation. This change could prevent or reduce the interactions between transcription factors and gene sequences in this region and further inhibit gene transcription and reduce protein expression, thus affecting the normal biological function of cells [24, 25] For instance, Leiszter et al. found that hypermethylation of the three CpG sites in *C5ORF66-AS1* downregulated its expression by preventing sp1 binding to these CpG sites [21]. Thus, we proposed that the *SST* methylation may interact with the expression in this way. We used bioinformatic analysis to predict that the differentially methylated CpG sites in *SST* common to all three tumor types (+42 and +44) may be binding sites for the transcription factors *RHOXF1*, *ETS1*, *GSC*, *GSC2*, *DPRX*, *OTX1*, and *OTX2*. The *SST* CpG sites +127 and +129 are the binding sites for the transcription factors *EBF1* and *NR2C2. ETS1* [26] and *OTX1* [27] have been verified to be related to the progression of GC. We speculated that during the malignant transformation of digestive tract epithelial cells, an abnormally high methylation of one or more specific CpG sites in the *SST* 1stExon region may inhibit *SST* transcription by inhibiting transcription factor binding, causing the occurrence and development of cancers. This hypothesis needs to be confirmed by in-depth molecular biology experiments.

Studies have shown that abnormal DNA methylation could be used as a diagnostic biomarker. Grutzmann et al. reported that methylation of *SEPT9* in plasma had a high SEN (72%) and SPE (90%) for diagnosing colorectal cancer [28]. *HOXA9* was found to be differentially methylated in patients with hepatic cancer compared with healthy people, with an SEN of 73.3% and an SPE of 97.1% [29]. A panel of five DNA methylation markers (*FER1L4*, *ZNF671*, *ST8SIA1*, *TBX15*, and *ARHGEF4*) detected 74% of EC cancer patients with an overall specificity of 91% [30]. In addition, in recent years, pancancer studies have found that multiple tumors share the same cancer pathways and biomarkers. Ge et al. reported that genes in the ubiquitin pathway were generally upregulated in 33 types of tumors and played an important role in the development of cancer [31]. Ding et al. identified 7 CpG sites that could effectively distinguish 12 major tumors in the TCGA database [32]. Therefore, it is possible to find biomarkers for the diagnosis and treatment of pandigestive tract cancers. In our study, we performed a combined analysis of the diagnostic efficacy of the *SST* methylation-specific CpG sites in GIT cancers. Finally, we established diagnostic models for combined and individual GIT cancers, and the results suggested that the *SST* methylation might be a potential new marker significantly associated with pandigestive cancers.

There are some limitations in our study. First, this was a single-center study, and the sample size was limited. In the future, multicenter studies are needed. Second, the sensitivity and specificity of the *SST* methylation for diagnosing pandigestive cancers need to be improved, and we hope to combine *SST* methylation with other biomarkers in the future. Moreover, in the future, we hope to detect differentially methylated CpG sites in *SST* in plasma samples or serum samples and to further evaluate the value and feasibility of the *SST* methylation as a noninvasive and early diagnostic marker for pandigestive cancers.

In summary, our results showed that the site-specific hypermethylation of *SST* 1stExon increased the risks of GIT cancers and might promote tumorigenesis and cancer progression by inhibiting gene transcription. In the future, the *SST* site-specific methylation may serve as a potential predictive biomarker for pan-GIT cancers.

## Figures and Tables

**Table 1:** Cohypermethylation CpG sites in GIT cancers.

Tumor	Hypermethylated CpG sites
EC	+18, +34, +42, +44, +92, +94, +100, +116, +127, +129, +138, + 148
GC	+18, +42, +44, +94, +100, +127, +129
CRC	+18, +34, +42, +44, +92, +94, +97, +100, +116,+127, +129
EC + GC + CRC	+18, +42, +44, +94, +100, +127, +129
EC + GC	+18, +42, +44, +94, +100, +127, +129
GC + CRC	+18, +42, +44, +94, +100, +127, +129
EC + CRC	+18, +34, +42, +44, +92, +94, +100, +116,+127, +129

**Table 2:** Correlation of methylation of SST and clinical phenotypes.

Cancer	Parameter	Group	*n*	18	25	34	42	44	85	92	94	97	100	116	127	129	138	148	AVG
EC	Lymphatic metastasis	Positive	25	0.497	0.654	0.71	0.311	0.405	0.858	0.617	0.749	0.798	0.265	0.405	0.828	0.969	0.497	0.788	0.513
		Negative	17																
	Vascular tumor emboli	Positive	9	0.718	0.976	0.786	0.587	0.414	0.249	0.526	0.651	0.303	0.171	0.303	0.021	0.038	0.075	0.098	0.224
		Negative	33																
	Depth of infiltration	Serosa	26	0.141	0.451	0.92	0.201	0.268	0.041	0.478	0.668	0.099	0.035	0.094	0.4	0.51	0.316	0.355	0.641
		Muscular	13																
		Mucosal and submucosal	3																
	Differentiation	Poorly	5	0.014	0.17	0.15	0.381	0.381	0.827	0.937	0.897	0.803	0.977	0.195	0.289	0.143	0.247	0.346	0.289
		Moderately	21																
		High	16																

GC	Lymphatic metastasis	Positive	64	0.471	0.013	0.073	0.257	0.578	0.339	0.821	0.804	0.91	0.958	0.566	0.869	0.848	0.487	0.301	0.924
		Negative	27																
	Vascular tumor emboli	Positive	57	0.961	0.993	0.085	0.608	0.426	0.348	0.724	0.931	0.879	0.583	0.688	0.737	0.831	0.7	0.403	0.974
		Negative	34																
	Depth of infiltration	Serosa	79	0.472	0.662	0.743	0.508	0.347	0.097	0.485	0.668	0.284	0.517	0.309	0.479	0.363	0.295	0.265	0.356
		Muscular	9																
		Mucosal and submucosal	3																
	Differentiation	Poorly	75	0.608	0.658	0.811	0.725	0.667	0.119	0.775	0.991	0.551	0.884	0.446	0.499	0.462	0.349	0.364	0.685
		Moderately	12																
		High	4																

CRC	Lymphatic metastasis	Positive	38	0.658	0.56	0.939	0.81	0.923	0.758	0.721	0.973	0.758	0.651	0.21	0.272	0.321	0.285	0.06	0.63
		Negative	42																
	Vascular tumor emboli	Positive	11	0.917	0.273	0.743	0.939	0.994	0.227	0.534	0.905	0.85	0.917	0.553	0.66	0.691	0.463	0.796	0.917
		Negative	69																
	Depth of infiltration	Serosa	59	0.581	0.891	0.267	0.184	0.102	0.9	0.13	0.025	0.184	0.083	0.119	0.058	0.253	0.207	0.227	0.173
		Muscular	21																
		Mucosal and submucosal	0																
	Differentiation	Poorly	25	0.76	0.36	0.53	0.289	0.151	0.519	0.24	0.081	0.351	0.271	0.426	0.139	0.497	0.647	0.334	0.157
		Moderately	40																
		High	15																

## Data Availability

The data generated in this study are available within the article and its supplementary data files. Methylation data analyzed in this study were obtained from The Genome Cancer Atlas (TCGA) database, which were downloaded from UCSC Xena (https://xenabrowser.net/datapages).

## Supplementary Materials

Supplementary 1 Supplementary Table S1: CpG sites in SST DMR.

Supplementary 2 Supplementary Table S2: SST Methylation in EC.

Supplementary 3 Supplementary Table S3: SST Methylation in GC.

Supplementary 4 Supplementary Table S4: SST Methylation in CRC.

Supplementary 5 Supplementary Table S5: Correlation between SST 1stExon methylation and SST expression in GIT cancers.
