# Supplementary material for: Site-Specific Hypermethylation of SST 1stExon as a Biomarker for Predicting the Risk of Gastrointestinal Tract Cancers
Source: Dis Markers. 2022 Feb 12;2022:4570290. doi: 10.1155/2022/4570290 (PMC8886765; doi:10.1155/2022/4570290)
Supplement: Supplementary 1 — Supplementary Table S1: CpG sites in SST DMR. [file 4570290.f1.docx]

**Supplementary Table S1: CpG sites in *SST* DMR**

| **Number** | **Cg site** | **Strand** | **Type** | **feature** | **cgi** |
| --- | --- | --- | --- | --- | --- |
| 1 | cg12146673 | F | I | Body | shore |
| 2 | cg05121480 | F | II | Body | shore |
| 3 | cg00481951 | R | II | Body | shore |
| 4 | cg00457403 | R | II | Body | shore |
| 5 | cg25478614 | R | II | Body | shore |
| 6 | cg15347189 | R | I | 1stExon | island |
| 7 | cg16927040 | R | I | 1stExon | island |
| 8 | cg02164046 | F | I | 1stExon | island |
| 9 | cg13206017 | F | II | TSS200 | shore |
| 10 | cg07120369 | F | II | TSS200 | shore |
