# Supplementary material for: Site-Specific Hypermethylation of SST 1stExon as a Biomarker for Predicting the Risk of Gastrointestinal Tract Cancers
Source: Dis Markers. 2022 Feb 12;2022:4570290. doi: 10.1155/2022/4570290 (PMC8886765; doi:10.1155/2022/4570290)
Supplement: Supplementary 2 — Supplementary Table S2: SST Methylation in EC. [file 4570290.f2.docx]

**Supplementary Table S2: SST Methylation in EC**

| **CpG site** | **Mean±Std** | | **P value** |
| --- | --- | --- | --- |
|  | **EC** | **EN** |  |
| 18 | 0.457±0.157 | 0.317±0.105 | <0.001 |
| 25 | 0.495±0.136 | 0.45±0.132 | 0.129 |
| 34 | 0.521±0.154 | 0.416±0.131 | 0.003 |
| 42 | 0.53±0.15 | 0.438±0.143 | 0.004 |
| 44 | 0.541±0.158 | 0.438±0.146 | 0.002 |
| 85 | 0.483±0.121 | 0.469±0.117 | 0.565 |
| 92 | 0.72±0.098 | 0.668±0.117 | 0.032 |
| 94 | 0.664±0.125 | 0.57±0.136 | 0.002 |
| 97 | 0.575±0.129 | 0.538±0.127 | 0.188 |
| 100 | 0.624±0.111 | 0.568±0.121 | 0.017 |
| 116 | 0.781±0.095 | 0.701±0.118 | 0.001 |
| 127 | 0.808±0.09 | 0.703±0.126 | <0.001 |
| 129 | 0.827±0.091 | 0.708±0.131 | <0.001 |
| 138 | 0.865±0.067 | 0.796±0.115 | 0.001 |
| 148 | 0.839±0.083 | 0.761±0.116 | 0.001 |
| AMR | 0.649±0.078 | 0.569±0.107 | <0.001 |

**P value:** the difference of SST methylation in EC and EN using Student’s t-test.

**EN:** Tumor-adjacent noncancerous tissues of EC
