# Supplementary material for: Site-Specific Hypermethylation of SST 1stExon as a Biomarker for Predicting the Risk of Gastrointestinal Tract Cancers
Source: Dis Markers. 2022 Feb 12;2022:4570290. doi: 10.1155/2022/4570290 (PMC8886765; doi:10.1155/2022/4570290)
Supplement: Supplementary 3 — Supplementary Table S3: SST Methylation in GC. [file 4570290.f3.docx]

**Supplementary Table S3: SST Methylation in GC**

| **CpG site** | **Mean±Std** | | **P value** |
| --- | --- | --- | --- |
|  | **GC** | **GN** |  |
| 18 | 0.416±0.116 | 0.359±0.111 | <0.001 |
| 25 | 0.494±0.097 | 0.482±0.12 | 0.404 |
| 34 | 0.51±0.102 | 0.486±0.089 | 0.098 |
| 42 | 0.556±0.123 | 0.492±0.091 | <0.001 |
| 44 | 0.545±0.144 | 0.489±0.101 | 0.002 |
| 85 | 0.479±0.122 | 0.474±0.091 | 0.734 |
| 92 | 0.678±0.11 | 0.669±0.085 | 0.551 |
| 94 | 0.62±0.132 | 0.576±0.099 | 0.009 |
| 97 | 0.58±0.138 | 0.547±0.1 | 0.061 |
| 100 | 0.602±0.132 | 0.568±0.106 | 0.041 |
| 116 | 0.715±0.088 | 0.69±0.088 | 0.061 |
| 127 | 0.744±0.103 | 0.705±0.097 | 0.009 |
| 129 | 0.765±0.104 | 0.711±0.105 | 0.001 |
| 138 | 0.825±0.08 | 0.804±0.086 | 0.102 |
| 148 | 0.788±0.087 | 0.77±0.091 | 0.186 |
| AMR | 0.622±0.09 | 0.588±0.079 | 0.008 |

**P value:** the difference of SST methylation in GC and GN using Student’s t-test.

**GN:** Tumor-adjacent noncancerous tissues of GC
