# Supplementary material for: Site-Specific Hypermethylation of SST 1stExon as a Biomarker for Predicting the Risk of Gastrointestinal Tract Cancers
Source: Dis Markers. 2022 Feb 12;2022:4570290. doi: 10.1155/2022/4570290 (PMC8886765; doi:10.1155/2022/4570290)
Supplement: Supplementary 4 — Supplementary Table S4: SST Methylation in CRC. [file 4570290.f4.docx]

**Supplementary Table S4: SST Methylation in CRC**

| **CpG site** | **Mean±Std** | | **P value** |
| --- | --- | --- | --- |
|  | **CRC** | **CRN** |  |
| 18 | 0.425±0.126 | 0.374±0.104 | 0.005 |
| 25 | 0.495±0.097 | 0.475±0.102 | 0.182 |
| 34 | 0.537±0.105 | 0.488±0.071 | 0.001 |
| 42 | 0.607±0.119 | 0.526±0.053 | <0.001 |
| 44 | 0.614±0.122 | 0.529±0.055 | <0.001 |
| 85 | 0.518±0.101 | 0.52±0.073 | 0.9 |
| 92 | 0.74±0.099 | 0.708±0.067 | 0.019 |
| 94 | 0.698±0.116 | 0.62±0.081 | <0.001 |
| 97 | 0.646±0.122 | 0.576±0.075 | <0.001 |
| 100 | 0.691±0.111 | 0.623±0.067 | <0.001 |
| 116 | 0.765±0.098 | 0.722±0.067 | 0.002 |
| 127 | 0.774±0.094 | 0.73±0.069 | 0.001 |
| 129 | 0.799±0.095 | 0.753±0.063 | 0.001 |
| 138 | 0.838±0.076 | 0.822±0.067 | 0.111 |
| 148 | 0.804±0.096 | 0.793±0.066 | 0.325 |
| AMR | 0.663±0.083 | 0.617±0.042 | <0.001 |

**P value:** the difference of SST methylation in CRC and CRN using Student’s t-test.

**CRN:** Tumor-adjacent noncancerous tissues of CRC
