# Supplementary material for: Site-Specific Hypermethylation of SST 1stExon as a Biomarker for Predicting the Risk of Gastrointestinal Tract Cancers
Source: Dis Markers. 2022 Feb 12;2022:4570290. doi: 10.1155/2022/4570290 (PMC8886765; doi:10.1155/2022/4570290)
Supplement: Supplementary 5 — Supplementary Table S5: Correlation between SST 1stExon methylation and SST expression in GIT cancers. [file 4570290.f5.docx]

**Supplementary Table S5: Correlation between SST 1stExon methylation and *SST* expression in GIT cancers**

| **CpG site** | **r** | | | ***P* *** | | |
| --- | --- | --- | --- | --- | --- | --- |
|  | **EC** | **GC** | **CRC** | **EC** | **GC** | **CRC** |
| +18 | -0.063 | -0.363 | -0.23 | 0.57 | < 0.001 | 0.008 |
| +25 | -0.13 | -0.154 | -0.17 | 0.239 | 0.118 | 0.053 |
| +34 | -0.071 | -0.271 | -0.23 | 0.518 | 0.005 | 0.008 |
| +42 | 0.005 | -0.393 | -0.379 | 0.966 | < 0.001 | < 0.001 |
| +44 | 0.026 | -0.371 | -0.396 | 0.816 | < 0.001 | < 0.001 |
| +85 | 0.025 | -0.107 | 0.083 | 0.818 | 0.28 | 0.346 |
| +92 | -0.001 | -0.226 | -0.193 | 0.989 | 0.021 | 0.028 |
| +94 | -0.003 | -0.355 | -0.406 | 0.975 | < 0.001 | < 0.001 |
| +97 | 0.083 | -0.252 | -0.36 | 0.453 | 0.01 | < 0.001 |
| +100 | 0.048 | -0.211 | -0.361 | 0.663 | 0.031 | < 0.001 |
| +116 | -0.089 | -0.252 | -0.273 | 0.419 | 0.01 | 0.002 |
| +127 | -0.034 | -0.255 | -0.272 | 0.758 | 0.009 | 0.002 |
| +129 | -0.047 | -0.334 | -0.306 | 0.668 | 0.001 | <0.001 |
| +138 | 0.074 | -0.292 | -0.179 | 0.501 | 0.003 | 0.041 |
| +148 | 0.045 | -0.212 | -0.129 | 0.687 | 0.031 | 0.144 |
| AVG | -0.026 | -0.35 | -0.374 | 0.813 | < 0.001 | < 0.001 |

*P：P value of the Pearson’s correlation coefficient between methylation and expression.
